# Editorial: Predictive Modeling of Human Microbiota and Their Role in Health and Disease

**DOI:** 10.3389/fmicb.2021.782871

**Published:** 2021-11-30

**Authors:** Hyun-Seob Song, Stephen R. Lindemann, Dong-Yup Lee

**Affiliations:** ^1^Department of Biological Systems Engineering, University of Nebraska-Lincoln, Lincoln, NE, United States; ^2^Department of Food Science and Technology, Nebraska Food for Health Center, University of Nebraska-Lincoln, Lincoln, NE, United States; ^3^Department of Food Science, Whistler Center for Carbohydrate Research, Purdue University, West Lafayette, IN, United States; ^4^Department of Nutrition Science, Purdue University, West Lafayette, IN, United States; ^5^School of Chemical Engineering, Sungkyunkwan University, Suwon, South Korea

**Keywords:** microbial biomarkers, machine learning, microbial association networks, species and gene profiling, precision nutrition

The human microbiota—communities of microorganisms living in or on humans as their host—are deeply involved in various biological processes and functions in our body and play an important role in maintaining physiological and mental health. The criticality of human microbiota is often indicated by the fact that bacterial cells (>100 trillion cells) in our body outnumber human cells (Sender et al., 2016) and carry ~150 times more genes than the entire human genome (Ursell et al., 2014)—thus called the second genome (Grice and Segre, 2012). Unlike the first genome, which remains largely invariable after being inherited from parents, the second genome's content dynamically changes under a variety of conditions affected by diets, drugs, stress, injuries, and myriad lifestyle and environmental factors. Hence, understanding of how microbiota respond to those perturbations and how the outcomes influence human health is important for developing microbiome-mediated strategies to improve it.

Mathematical modeling of human microbiota and their interactions with host cells under various environmental conditions is indispensable in this regard. The effectiveness of mathematical models has been demonstrated through various applications, including inferring microbe-microbe, microbe-host, and microbe-host-diet interactions (Song et al., 2014; Li et al., 2019; Chowdhury and Fong, 2020), creating new insights into the role of human microbiota in health and disease state (Kumar et al., 2019), and proposing new engineering strategies for intervention (Sheth et al., 2016; Kessell et al., 2020). In addition to process-based models, data-driven modeling is also being popularly used (Marcos-Zambrano et al., 2021) and the combination of these complementary approaches is expected to significantly increase the scope of prediction (Kessell et al., 2020).

Despite promising progress over the past decade, we still lack a complete understanding of the relationships between microbiota (composition and function) and the host (health and disease) under perturbed conditions as well as in homeostasis, and consequently have a limited capability of predicting their interplays. Toward addressing those challenges and promoting new opportunities, this Research Topic collects nine research articles and one review that present the state-of-the-art modeling approaches in various areas of human microbiome research. Below we summarize the contributions from lead computational biologists under the following several categories: discovery of microbial biomarkers and signatures of diseases, quantitative assessment of the impact of bacteria on human health and their association with disease, improved profiling of species and genes in the microbiome, and incorporation of microbiome data with other clinical features for improved precision nutrition and medicine.

Human microbiota show compositional changes across conditions, including physiological states of the host in health and disease. Particular enrichments of microbes, if observed, can therefore be used as a biomarker of specific diseases. Those signatures could further be considered potential diagnostic and therapeutic targets. Several contributions in the Research Topic address this issue. To predict the risk for developing colorectal metachronous adenoma (MA) after surgical resection, Liu et al. developed a random forest (RF) model using the relative abundance of the gut microbial populations with or without the clinical risk factors. This work is based on the hypothesis that the composition of the gut microbiota before surgery was associated with the risk of developing MA and, therefore, could serve as a potential biomarker for MA. The resulting RF model identified *Escherichia*-*Shigella* and *Acinetobacter* as key microbiome biomarkers, although the accuracy of prediction was improved when linked with other clinical risk factors such as synchronous adenoma and body mass index. Kort et al. examined how the hypothesis on the use of gut microbiomes as a biomarker can be extended to identify the association of gut microbiota with language development of young children. Using data from rural 3-year-olds in Uganda, they developed regression models by accounting for all possible combinations of three or four species. This comprehensive survey of regression models of all subsets of species led to the identification of *Coprococcus eutactus*, an anaerobic butyrate-producing gut bacterium, as a major predictor of language development in children. In the study of non-small cell lung cancer patients treated with different cycles of osimertinib therapy, Cong et al. identified the shifts in microbial biomarkers between post- and pre-therapy. Through the analysis of intestinal microbial ecological networks constructed by random matrix theory methods, they also found the structure of microbial interaction networks became complicated by including more compact modules in response to osimertinib therapy. A knowledgebase system of the human colorectal cancer (CRC) microbiome constructed by Zhou et al. integrates complementary data and information to improve the predictive power of models in biomarker prediction. The web-based platform allows for systematic inquiry and comparison across different models or databases to identify microbial biomarkers through statistical analysis. The important goal of this platform is to facilitate diagnosis of CRC, identify key factors for clinical transformation, and contribute to the development of cost-effective screening strategies.

Beyond discovering microbiome biomarkers and signatures of disease, another central research challenge is to decipher direct linkages between microorganisms and specific disease types, e.g., through a data-driven network analysis or mechanistic modeling. Along this line, Lei and Wang proposed a new method that enables integrating two similarity-based networks of microorganisms and diseases through the known microbe-disease associations. The resulting integrative network of microbes and diseases may potentially create a new mechanistic understanding of microbe-disease associations that are previously unknown. Compared to existing approaches, the proposed method showed effectiveness in predicting microbe-disease association, as demonstrated through case studies of asthma, chronic obstructive pulmonary disease, and inflammatory bowel disease. A more mechanistic prediction of the bacterial impact on risk factors that may cause disease was made by Bourgin et al. In order to evaluate the impact of the microbial activity vs. host on the cholesterol cycle, they developed a whole-body human model of cholesterol metabolism by incorporating bacterial conversion of bile salts and cholesterol into the existing models that focus on host metabolism. Comprehensive simulations using the model showed that cholesterol conversion to bile salts is the main flux of cholesterol cycle, indicating that bacterial metabolism likely drives cholesterol regulation.

Further, maintenance of the community structure and function of human microbiomes by regulating the ecological balance of microbial populations is key, as disturbances are linked with negative outcomes on human physiological and psychological health. Maintaining a desirable ecological balance of populations in the human microbiome is important because alterations in their composition and function (i.e., dysbiosis) are linked with detrimental physiological and psychological impacts, and result in a wide array of disease conditions. The dynamic model developed by Dedrick et al. can serve as a useful tool to understand bacterial coexistence and stability. The authors constructed an *in silico* model of nasal microbiota composed of up to 20 isolates to predict how the community composition responds to the variation of pH fluctuations in amplitude or frequency. The simulation results showed no significant impact of temporal pH fluctuations on the species coexistence and composition. The numerical model also suggested cooperative interactions among member species that have low niche overlap as a potential mechanism for the observed robustness of nasal microbiota.

Development of predictive human microbiome models is facilitated by advanced gene sequencing technologies, including amplicon sequencing for bacterial composition profiling and shotgun sequencing for metagenomic analyses. As pointed out by Gwak and Rho, it is challenging to perform accurate taxonomic assignment at species level because the 16S rRNA sequences among species in the same genus are highly homologous or even identical. To improve the resolution, they reannotated inconsistent or mislabeled taxa in three major 16S rRNA databases and determined species-level taxonomy using a *k*-nearest neighbor algorithm and the consensus models constructed for each species. In the case studies using salivary and gut microbiome data, the proposed method successfully identified the variation in bacterial composition across different groups based on improved species-level profiling. Ma argued that metagenomic gene abundance data can be analyzed in a similar fashion to operational taxonomic unit analysis by viewing microbiomes as a community of genes, rather than species. Using Taylor's power law, this work analyzed the impact of obesity, inflammatory bowel disease, and diabetes on the human microbiota, highlighting the importance of a sound understanding of metagenomic heterogeneity for the success of personalized and precision medicine to treat the human microbiome-associated diseases.

Finally, one of the major goals in the human microbiome research is to fundamentally elucidate the complex interactions among diet, gut microbiome, and human health so that we have an improved capability of monitoring wellness states, treating diseases, designing food products, and administering health interventions. Eetemadi et al. provide a review on this issue by discussing a critical role of predictive computational models, particularly machine learning and artificial intelligence. They shared the state of dietary recommendation systems (RSs) and highlight the transition from population-wide to microbiome-aware RSs to provide users with personalized guidelines. They also discussed the details about three complementary approaches for realizing microbiome-aware RS, including knowledge-based, content-based, and collaborative filtering RSs.

The overarching goal of computational modeling of human microbiomes is to enhance our ability to predict their dynamics, association with human disease, and control points that can be used to shape microbiome composition and functions toward improved human health. Some of these control points may target the organisms themselves, others may shape their environments to accomplish these ends. The work contained in this Research Topic uses multiple techniques to provide increased insight into environment-human microbiome-health connections and to substantially advance the field of predictive human microbiome modeling.

## Author Contributions

All authors listed have made a substantial, direct, and intellectual contribution to the work and approved it for publication.

## Funding

D-YL received funding from the SMC-SKKU Future Convergence Research Program grant and the National Research Foundation of Korea (NRF) grant funded by the Korea government (MSIT) (No. 2020R1A2C2007192).

## Conflict of Interest

The authors declare that the research was conducted in the absence of any commercial or financial relationships that could be construed as a potential conflict of interest.

## Publisher's Note

All claims expressed in this article are solely those of the authors and do not necessarily represent those of their affiliated organizations, or those of the publisher, the editors and the reviewers. Any product that may be evaluated in this article, or claim that may be made by its manufacturer, is not guaranteed or endorsed by the publisher.
